# Novel (Q)SAR models for prediction of reversible and time-dependent inhibition of cytochrome P450 enzymes

**DOI:** 10.3389/fphar.2024.1451164

**Published:** 2025-02-12

**Authors:** Sadegh Faramarzi, Arianna Bassan, Kevin P. Cross, Xinning Yang, Glenn J. Myatt, Donna A. Volpe, Lidiya Stavitskaya

**Affiliations:** ^1^ Office of Clinical Pharmacology, Center for Drug Evaluation and Research, Food and Drug Administration, Silver Spring, MD, United States; ^2^ Instem Inc., Conshohocken, PA, United States

**Keywords:** CYP = cytochrome P450, reversible inhibition, time dependent inhibition, QSAR, SAR, computational model

## Abstract

The 2020 FDA drug-drug interaction (DDI) guidance includes a consideration for metabolites with structural alerts for potential mechanism-based inhibition (MBI) and describes how this information may be used to determine whether *in vitro* studies need to be conducted to evaluate the inhibitory potential of a metabolite on CYP enzymes. To facilitate identification of structural alerts, an extensive literature search was performed and alerts for mechanism-based inhibition of cytochrome P450 enzymes (CYP) were collected. Furthermore, five quantitative structure-activity relationship (QSAR) models were developed to predict not only time-dependent inhibition of CYP3A4, an enzyme that metabolizes approximately 50% of all marketed drugs, but also reversible inhibition of 3A4, 2C9, 2C19 and 2D6. The non-proprietary training database for the QSAR models contains data for 10,129 chemicals harvested from FDA drug approval packages and published literature. The cross-validation performance statistics for the new CYP QSAR models range from 78% to 84% sensitivity and 79%–84% normalized negative predictivity. Additionally, the performance of the newly developed QSAR models was assessed using external validation sets. Overall performance statistics showed up to 75% in sensitivity and up to 80% in normalized negative predictivity. The newly developed models will provide a faster and more effective evaluation of potential drug-drug interaction caused by metabolites.

## 1 Introduction

Cytochrome P450 (CYP) enzymes are a family of heme containing enzymes that catalyze the oxidative metabolism of drugs, chemical carcinogens, steroids, and fatty acids (Guengerich, 2001). Drugs and other xenobiotics may inhibit or induce CYP enzymes, and therefore alter the metabolism of co-administered drugs. This phenomenon makes up the majority of drug-drug interaction (DDI) (Hansten et al., 2010). DDI has led to withdrawal of drugs such as mibefradil, terfenadine, bromfenac, cisapride, cerivastatin, *etc.*, from the market (Wienkers and Heath, 2005; Giacomini et al., 2007; O Nettleton and J Einolf, 2011). Adverse drug reactions from DDIs are also the fourth leading cause of death in the US (Borda et al., 1968; Costa, 1991).

There are at least 57 human CYP enzymes among which 12 have been reported to be involved in drug metabolism (Lewis and Ito, 2010). It was reported that 52% of small molecule drugs approved by the U.S. FDA in 2015–2020 were metabolized by CYP3A4, making it the major CYP subtype (Guengerich, 2022). CYP enzyme inhibition is generally categorized as reversible or irreversible. Mechanism-based inhibition (MBI) is a sub-category of irreversible inhibition, while reversible inhibition (RI) usually occurs when two or more molecules compete for binding to active or allosteric sites of a CYP enzyme. RI can also be non-competitive, where the inhibitor the inhibitor alters the active site of the enzyme so that the enzyme loses affinity for its substrate (Deodhar et al., 2020). MBI typically involves the conversion of a drug to a reactive metabolite(s), which may react with components of cellular proteins, DNA, and may even lead to enzyme destruction (Zhang and Tang, 2018). The MBI mechanism leads to a change in potency of CYP inhibitors that depends on *in vitro* incubation time or dosing period *in vivo*, and this change is referred to as time-dependent inhibition (TDI) (Riley et al., 2007). As a result, CYP inhibition presents a great risk in drug development process.

In 2020, FDA finalized a guidance to assist drug developers with evaluating the DDI potential of investigational drugs (www.fda.gov/media/134582/download). Specifically, the guidance provides a framework for conducting and interpreting *in vitro* studies for new drugs. Furthermore, the guidance states that *in vivo* DDI caused by metabolites may be possible even if the *in vitro* studies suggest that the parent drug alone will not inhibit any major CYP enzymes (www.fda.gov) (Sudsakorn et al., 2020). Therefore, the guidance recommends that drug developers consider evaluating metabolites *in vitro* for their inhibitory effects on a panel of CYP enzymes. Specifically, an *in vitro* CYP enzyme inhibition study is recommended “if the metabolite is (1) less polar than the parent drug and the area under the plasma concentration-time curve (AUC) of a metabolite is ≥25% of AUC of the parent or (2) if the metabolite is more polar than the parent drug and the AUC of metabolite is ≥ AUC of the parent drug”. In addition, a “lower cut-off value for the metabolite-to-parent AUC ratio may also be considered if a metabolite contains a structural alert for potential MBI” of CYP enzymes, since such inhibition carries a higher risk of causing drug interaction due to their prolonged inhibition effect.

Various computational approaches have been developed to assist with prioritization of drug candidates that have a low propensity for CYP inhibition. Most of QSAR models for enzyme inhibition are ligand-based *in silico* methods that predict the biological activities of drugs based on their 2D or 3D structures without knowing the 3D structure of the target protein or enzyme. Early QSAR models related reversible CYP inhibition of compounds to molecular properties such as octanol-water partition coefficient, polarizability, Taft steric parameter, and molecular volume (Hansch and Zhang, 1993; Gao and Hansch, 1996; Lewis et al., 2001; Hansch et al., 2004). However, subsequent 2D and 3D QSAR models included chemical structural descriptors. Examples of these models include CYP2D6 inhibition models using 26 aryloxypropanolamine compounds and CYP3A4 inhibition models (Roy and Roy, 2009). A similar approach was employed by Didziapetris et al. where baseline global QSAR models and local similarity-based corrections for over 800 compounds from various literature sources were used to predict CYP3A4 inhibition (Didziapetris et al., 2010). These models provide accuracies of 80%–83% for the internal test set and 75%–77% for the external test set. Among the most interesting findings from this study was that the presence of hydrophobic residues in a compound favored CYP3A4 inhibition while strong acidic or basic groups reduced inhibition probability. Another noticeable model was built by Cheng et al. (2011), using a combination of classifiers to predict direct inhibition of 5 CYP enzymes. Although the dataset included over 24,700 compounds, compounds with IC_50_ values in the range of 10–57 µM have been classified as equivocal and were excluded to avoid uncertainty during model development, leaving 15,744 compounds for the training set. More recently, a model to predict inhibition or induction of 5 common CYP enzymes has been made available by Rudik et al. (2022). These models calculated pIC_50_ values (negative log of the IC50 value) and the largest training set consists of 16,997 compounds (5,702 positive). However, these models do not discriminate between RI and MBI.

In addition to traditional QSAR models, experimental screening methods have been employed to gain insight into CYP inhibition mechanisms. In a report by Gonzalez et al. (2021), ∼5,000 compounds were screened for inhibition of CYP2C9, CYP2D6, and CYP3A4 using a luminescence-based cytochrome P450 assay. The resulting data were stratified in random forest and multi-task deep neural networks to construct QSAR models. A balanced accuracy of approximately 0.7 was achieved using the best model. Additionally, Veith et al., have screened 17,143 compounds against five recombinant CYP isozymes (1A2, 2C9, 2C19, 2D6 and 3A4) using an *in vitro* bioluminescent assay (Veith et al., 2009). Experimental data were used to interrogate inhibition activities of functional groups and molecular features. Finally, there have been molecular docking simulations, homology modeling, and molecular dynamics simulations of CYP enzymes that are outside of the scope of the current study.

Earlier QSAR models that relate CYP enzyme inhibition to molecular properties of inhibitors often use relatively small training sets which limits their applicability in a regulatory setting. More recent neural network models use larger datasets and offer higher accuracies compared to traditional models. However, they use a “black box” approach and therefore, identification of structural features responsible for the enzyme inhibition is challenging, if not impossible. As mentioned earlier, another critical shortcoming of most of the previous models is that they do not discriminate between RI and TDI. In addition, 3D-QSAR models are usually constructed under the assumption that the enzyme binding mode for all compounds is the same which may not be true (Ekins et al., 1999; Wang et al., 2000; Lu et al., 2001; Galetin et al., 2003; Ekroos and Sjögren, 2006). Furthermore, some of QSAR models are proprietary and therefore cannot be assessed or reproduced due to unavailability of the training set data.

Due to these limitations, the present study focused on developing QSAR models using publicly available and chemically diverse data sets for TDI of CYP3A4 and RI of CYP3A4, CYP2C9, CYP2C19, and CYP2D6 enzymes. In addition, structural features that are responsible for enzyme inhibition have been identified. Overall, the newly constructed models can be used to identify molecular fragments that are responsible for CYP inhibition.

## 2 Materials and methods

### 2.1 QSAR model database

All training set data used to construct CYP inhibition QSAR models were comprised of non-proprietary data harvested from public data sources including BindingDB (www.bindingdb.org), Google Scholar, PubMed, and US Patents. All references are provided in the Supplementary Table S1. RI data were collected for the four most common CYP enzymes, 3A4, 2C9, 2C19 and 2D6 and TDI data was harvested for 3A4. Although RI data were collected for CYP1A2, CYP2B6, and CYP2C8 as well as TDI data for CYP1A2, CYP2B6, CYP2C8, CYP2C9, and CYP2C19, the databases were small and did not yield viable models (data not shown).

The RI potential of a drug was measured using the concentration of the drug required to reduce the enzymatic activity of the enzyme towards its known substrate to half of its normal value, *IC*_*50*_, or RI constant, *K*_*i*_. However, the current FDA guidance recommends assessment of *R*_*1*_ using the following Equation 1:

$$R_{1}=1+\frac{I_{\max,u}}{K_{i,u}}$$ (1)

where $I_{\max,u}$ is the maximal unbound plasma concentration of the interacting drug at steady state and $K_{i,u}$ is the unbound inhibition constant determined *in vitro* (www.fda.gov/media/134582/download). In the present study, RI data were collected for *IC*_*50*_, *K*_*i*_, and *R*_*1*_ measurements, where available.

For irreversible inhibition, *IC*_*50*_ from direct inhibition is usually compared to the IC_50_ after preincubation of the enzyme with a cofactor. A decrease in *IC*_*50*_ upon preincubation indicates TDI (*i.e*., IC_50_ shift). Other common constants used in assessment of TDI were the maximal inactivation rate constant (*k*_*inact*_), inactivator concentration that yields half of the maximum inactivation rate (*K*_*I*_), and the first order rate constants for loss of CYP activity (*k*_*obs*_) defined by the following Equation 2 (Orr et al., 2012):

$$k_{obs}=k_{obs,\left[I\right]=0}+\frac{k_{inact}\left[I\right]}{K_{I}+\left[I\right]}$$ (2)

where $k_{obs,\left[I\right]=0}$ is $k_{obs}$ in the absence of the substrate, and $\left[I\right]$ is the concentration of the inactivator. In addition, the FDA guidance recommends assessment of *R*_*2*_, which is defined as Equations 3, 4:

$$R_{2}=\frac{k_{obs}+k_{\deg}}{k_{\deg}}$$ (3)

where

$$k_{obs}=\frac{k_{inact}\times 50\times I_{\max,u}}{K_{I,u}+50\times I_{\max,u}}$$ (4)

and $k_{\deg}$ is the apparent first-order degradation rate constant of the affected enzyme.

A binary scoring system was used for modeling purposes where a score of “1” was assigned to all chemicals classified as inhibitors or inactivators and “0” was used to denote non-inhibitor or non-inactivator. The experimental criteria used to classify CYP inhibition is presented below, in Table 1. The thresholds used to define reversible inhibition were obtained from available literature (Rao et al., 2000; Rudik et al., 2022), while the thresholds used for R_1_, IC_50_ fold shift and R_2_ were obtained from the FDA DDI guidance. Curated datasets are available in Supplementary Table S1. Overall, there are 10,129 unique chemicals in the training sets.

**TABLE 1:** Thresholds for identification of reversible and time-dependent inhibitors.

Inhibition type		Classification	
	Parameter	Inhibitor (scored as “1”)	Non- inhibitor (scored as “0”)
Reversible Inhibition (RI)	IC_5o_ (µM)	≤10	>10
	K_i_ (µM)	≤10	>10
	R_1_	≥1.02	<1.02
Time dependent Inhibition (TDI)	IC_50_ fold shift	≥1.5	<1.5
	Change in inhibition (%)	≥20	<20
	k_obs_ (min^-1^)	≥0.01	<0.01
	R_2_	≥1.25	<1.25

### 2.2 Chemical structure curation

The chemical structures were obtained from BindingDB, SciFinder^®^, and PubChem databases in structure data file (SDF) format. Inorganic chemicals, noble gases, mixtures, single atoms, metals, and high molecular weight compounds (MW ≥ 1800) were excluded from the training set. For salts, the neutralized free forms were used. Manual inspection was performed to ensure the chemicals, their associated data and references were accurately recorded.

### 2.3 SAR profiler literature curation

Structural features that are susceptible to bioactivation have been outlined in the literature, where collections of such features have been discussed and reviewed. In the present work, key publications (Table 2) have been analyzed to collate a set of structural features associated with bioactivation potentially leading to MBI of CYP enzymes. More specifically, a list of structural alerts associated with the formation of reactive metabolites was compiled together with the information on available evidence on their association with MBI including annotations on the proposed mechanisms underlying bioactivation and examples. These alerts were encoded into a Leadscope alert set to profile potential CYP inhibition and is referred to as the Structure-Activity Relationship (SAR) Profiler.

**TABLE 2:** Key publications that have been analyzed to collate structural alerts associated with bioactivation potentially leading to MBI of CYP enzymes.

Publication details	References
Common substructures causing mechanism-based inactivation of cytochromes P450 (e.g., furans and thiophenes, dichloro- and trichloro-ethylenes)	Fontana et al. (2005)
Diverse functional motifs used in drug-design that potentially undergo bioactivation	Kalgutkar et al. (2005)
Cytochrome P450 inhibition by different chemical classes	Correia (2005)
Bioactivation potential of organic functional groups	Kalgutkar and Soglia (2005)
Known structure-activity relationships for P450 inactivation	Kalgutkar et al. (2007)
List of organic functional groups susceptible to bioactivation	Kalgutkar (2008)
Analysis of drugs based on known structural alerts associated with bioactivation	Stepan et al. (2011)
Well-known toxicophores associated with bioactivation leading to reactive metabolite formation	Kalgutkar et al. (2012)
Functional groups associated with mechanism-based inactivation of CYP enzymes	Orr et al. (2012)
Analysis of structural alerts associated with reactive metabolite formation	Kalgutkar and Dalvie (2015)
Structural alerts for P450 inactivation	Yu et al. (2015)
Analysis of the structural alert/reactive metabolite concept	Kalgutkar (2020)

The analysis was also enriched with an additional literature search to complement the collected information especially on underlying bioactivation mechanisms (Bolleddula et al., 2014; Claesson and Minidis, 2018; Cerny et al., 2020; Kalgutkar and Driscoll, 2020; Zhang et al., 2020).

### 2.4 Leadscope software

Leadscope Enterprise (LS) version 3.9 (Instem Inc., USA) was used to construct binary QSAR models. LS is a data mining, visualization, and advanced informatics application with the capability to build and apply 2D QSAR models. The software program was acquired and used under Research Collaboration Agreements between FDA/CDER and the software provider. All training set structures were imported into LS and fingerprinted using a set of 27,142 pre-defined medicinal chemistry structural features. A small predictive subset of these features was used to construct the model. Structural scaffolds were generated for the CYP inhibition models using the following criteria: 1) a minimum of three compounds per scaffold; 2) a minimum of six atoms per scaffold; 3) no restriction on the maximum number of rotatable bonds; and 4) a minimum absolute difference between the mean activity of the subset of compounds having that feature and the mean activity of the full set (Roberts et al., 2000). Molecular properties such as molecular weight, number of rotatable bonds, number of hydrogen bond donors, number of hydrogen bond acceptors, Lipinski score, AlogP (logarithm of 1-octanol/water partition coefficient), polar surface area, and atom count were calculated and used for building models.

Highly predictive features and the corresponding helper features were identified in the feature editor for retention while weakly predicted features were removed using Z-score, frequency, precision and mean activity as discriminating parameters (Roberts et al., 2000). Additional inspection was manually performed, and redundant or highly correlated features were removed.

For each model, cross-validation was performed 10 times using a 10% × 10% leave-many-out (LMO) method. This method randomly selects 10% of the training set for testing and reconstructs a reduced model using the remaining 90% of the compounds and recalculates the descriptor weights. This process was repeated 10 times to ensure that all the compounds present in the training set were predicted ten times. The average predicted values were used to generate a classic 2 × 2 contingency table containing counts of true positives (TP), true negatives (TN), false positives (FP), and false negatives (FN) and to evaluate predictive performance. Statistics such as sensitivity [TP/(TP + FN)], specificity [TN/(TN + FP)], positive predictivity [TP/(TP + FP)], negative predictivity [TN/(TN-FN)], and accuracy [(TP + TN)/(TP + TN + FP + FN)] were calculated as described by Cooper et al., 1979. Chemicals classified as out-of-domain (OOD) and equivocal were excluded from Cooper statistic calculations. Coverage was calculated as the percentage of all chemicals screened for which a prediction could be made (OOD results do not constitute a prediction).

A classification threshold was determined by varying the positive cutoff probability thresholds for equivocal results and analyzing the resulting Cooper statistics. The optimal probability range for indeterminate predictions for the models were identified to be 0.4 to 0.6. Predictions that are above the 0.6 probability cutoff were classified as positive, while predictions below 0.4 were classified as negative. A chemical was treated as OOD if it did not contain any structural model features or showed a lack of similarity to the training set compounds (at least 30% similarity to a single training set compound was required).

Additionally, structural features associated with MBI as proposed in the key publications (Table 2) were encoded in a preliminary version of a computerized expert-rule based profiler. Whereas the literature-based structural alerts are in general broadly defined and often liable to different interpretations, the computerized expert system had to specify all the details of the chemical environment associated with a given alerting chemical feature. A set of initial structural alerts were encoded and then subjected to a fingerprinting analysis. This methodology was developed to identify SAR knowledge across a series of proprietary chemicals databases containing relevant data without revealing any potentially confidential information on the individual chemicals (Ahlberg et al., 2016; Amberg et al., 2019). More specifically, the encoded structural alerts preliminarily defined from the literature curation were complemented with additional probing structural features (i.e., chemical features covering additional chemical space as implemented by Leadscope (Ahlberg et al., 2016)). The alerts and the additional features were matched against a proprietary database of chemicals where it was known whether a given chemical record was a CYP3A4 mechanism-based inhibitor or not. Such proprietary datasets were assembled by industry and made available to Leadscope through the fingerprinting analysis without disclosing the structures of the chemicals. Counts of the number of positive and negative hits (i.e., chemicals which are mechanism-based inhibitors and chemicals which are not mechanism-based inhibitors, respectively) from the proprietary database matching each substructure (i.e., the alerts and the additional features) were calculated using the Leadscope software. Each chemical feature (i.e., substructure) was then associated with a count of positives and negatives.

The results of the fingerprinting analysis were used to manually refine the structural alerts that were initially encoded from literature curation. This means that the resulting refined computerized expert-rule based profiler included a set of MBI alerts where the chemical environment of the alerts was manually revised based on the fingerprinting exercise as compared to the very general features defined in the literature. These alerts were then qualified using the 3A4 TDI dataset of 620 compounds. More specifically, each MBI alert was marked as predictive or indeterminate based on available evidence; an alert was marked as indeterminate if there were 3 or less examples from the CYP3A4 TDI dataset (i.e., chemicals from the CYP3A4 TDI dataset matching the alert of interest), or alert was evaluated not to be statistically significant (i.e., binomial distribution probability greater than 0.85). Expert review of the assignment was also carried out to revise it based on visual inspection of the chemicals in the CYP3A4 TDI dataset bearing the specific alert.

### 2.5 External validation

The predictive performance of the CYP models was assessed using external validation sets comprised of approved drugs harvested from proprietary FDA applications and publicly available data sources including BindingDB (www.bindingdb.org), Google Scholar, PubMed, and US Patents. Similar to the training sets, the criteria in Table 1 were used to classify the compounds in the validation sets. For consensus predictions using the SAR profiler and QSAR models, a positive prediction from the SAR profiler was used to justify an overall positive prediction when QSAR prediction was equivocal only.

## 3 Results

### 3.1 Database overview

**FIGURE 1:**
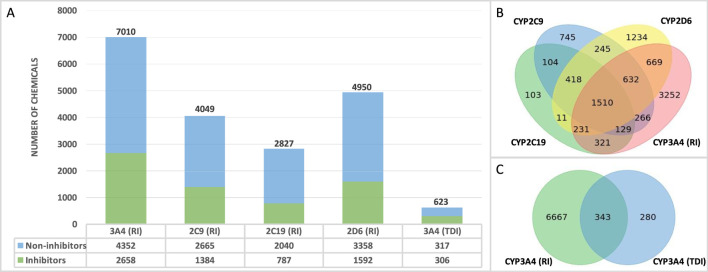
Database overview **(A)** Number of non-proprietary chemicals in each database. **(B)** Number of chemicals tested in one or more CYP enzyme. **(C)** Number of overlapping chemicals in the CYP3A4 (RI) and CYP3A4 (TDI) databases.

The final QSAR modeling database set contains 10,129 unique chemicals compiled from public sources. The number of chemicals in each individual set is shown in Figure 1A. Each training set contains less inhibitors as compared to non-inhibitors with 38%, 34%, 28%, 32%, and 49% positive chemicals in CYP3A4 (RI), CYP2C9, CYP2C19, CYP2D6, and CYP3A4 (TDI), respectively. Figure 1B shows the number of chemicals present in one or more reversible CYP inhibition training sets. Overall, there are 151,058 chemicals present in all four RI datasets. Of these chemicals, 983 exhibit the same effect on all four CYP enzymes (with only 78 being inhibitors), reflecting the diversity of the binding sites of those enzymes. In addition, a total of 343 compounds were found to be in common between CYP3A4 RI and CYP3A4 TDI training sets (Figure 1), showing 59% concordance.

**FIGURE 2:**
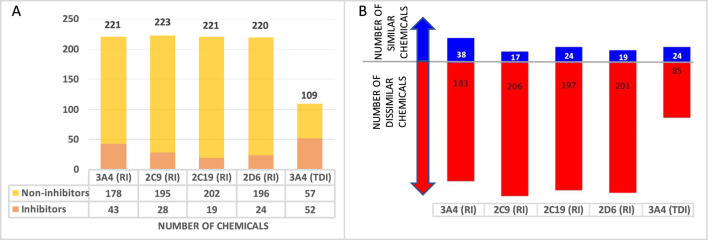
External validation database overview **(A)** Number of chemicals in each database. **(B)** Number of structurally similar (blue) and dissimilar (red) drugs in the external validation set when compared to the training set.

Additionally, proprietary data for CYP inhibition were harvested for a total of 231 approved drugs and 33 non-proprietary chemicals to use for external validation. The number of chemicals in each individual set is presented in Figure 2A. A structure similarity analysis between the training and validation sets revealed that the external validation sets are 83%–98% dissimilar when a similarity threshold of 70% is applied (Figure 2B).

### 3.2 Molecular descriptor analysis

**FIGURE 3:**
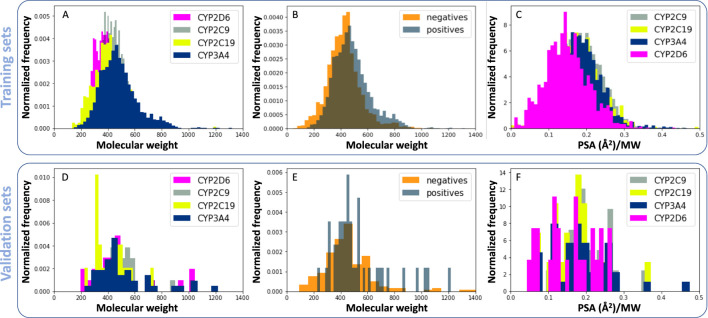
Relationship between molecular descriptors and reversible CYP inhibition. **(A, D)** Normalized histograms for molecular weight of CYP inhibitors. **(B, E)** Molecular weight of CYP3A4 inhibitors (positives) compared to non-inhibitors (negatives). **(C, F)** Polar surface area (PSA) of reversible CYP inhibitors divided by their molecular weight. Panels A, B and C were generated using training sets and D, E, and F were generated using validation sets.

The relationship between molecular descriptors and RI databases is presented in Figure 3. As seen in previous studies (Lewis and Ito, 2010), CYP3A4 may be inhibited by large molecules, due to its large cavity size, when compared to other CYP enzymes as shown Figure 3A. The molecular weight distribution of CYP3A4 inhibitors (positives) is also slightly higher than non-inhibitors (negatives, Figure 3B). Another interesting finding is that CYP2D6 inhibitors are on average less polar than the rest of the enzymes, specifically CYP3A4 (Figure 3C). Docking studies identify a hydrophobic binding pocket for CYP2D6 (VandenBrink et al., 2012), which is consistent with lower polar surface area (PSA) of its substrates (Figure 3C). Similar trends were observed for validation sets, despite their small size (Figures 3D–F).

### 3.3 Development of QSAR models

In the present study, two modeling approaches were utilized to construct RI and TDI QSAR models. The CYP3A4 TDI models were constructed using a single model approach, while reversible CYP inhibition models were constructed using average-modeling approach to obtain an optimal active-to-inactive ratio. For the average-modeling approach, submodels were created using subsets of the training set with optimal active-to-inactive ratio such that all of the compounds are present in at least one submodel. When the average model is applied, each submodel predicts a value, and the average of all of the predicted values is reported. The final models contained a total of 524, 545, 682, 592 and 361 features in the CYP3A4 (RI), CYP2C9, CYP2C19, CYP2D6, and CYP3A4 (TDI), respectively. The structural features with highest Z-scores for each individual model is presented in Table 3.

**TABLE 3:** Top structural features for CYP inhibition.

CYP3A4	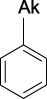 toluene		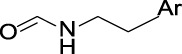 *N*-ethyllformamide	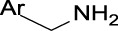 Amino arylmethane	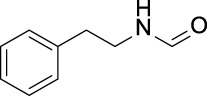 *N*-phenethylformamide	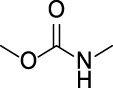 carbamate
	Z-Score	13	13	13	12	12
	Frequency	3,185	311	1,561	288	204
CYP2C9	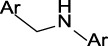 aryl-arylamino ethane		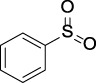 sulfonylbenzene	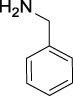 Aminomethyl-benzene	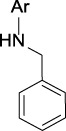 Aryl-aminomethyl-benzene	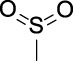 sulfonyl
	Z-Score	11	10	9	8	7
	Frequency	200	586	476	133	799
CYP2C19	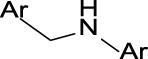 aryl-arylamino ethane		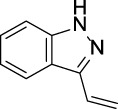 3-vinyl-1*H*-indazole	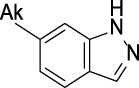 6-alkyl-1*H*-indazole	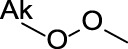 alkylperoxide	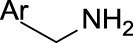 amino arylmethane
	Z-Score	9	8	8	8	7
	Frequency	137	27	24	25	546
CYP2D6	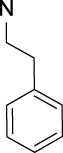 Aminomethyl-benzene		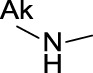 secondary alkylamine	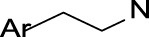 Amino-arylethane	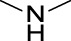 secondary amine	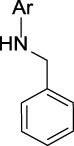 Alkyl-aminomethyl-benzene
	Z-Score	18	16	13	13	13
	Frequency	864	1,143	312	1,324	673
CYP3A4 TDI	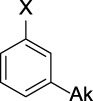 alkyl halobenzene		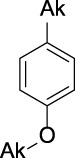 Alkoxy-4-alkylbenzene	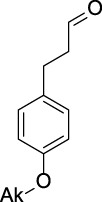 Alkoxy-4-(oxopropyl)-benzene	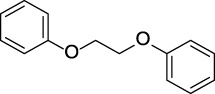 1,2-diphenoxyethane	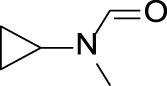 *N*-cyclopropyl-*N*-methylforamide
	Z-Score	8	6	6	6	5
	Frequency	114	83	41	33	57

### 3.4 Development of SAR profiler for MBI

The literature analysis and the fingerprinting exercise led to the definition of 58 MBI structural alerts (see Supplementary Table S2 for the complete list of alerts). Each alert is built using different rules covering a specific chemical environment to identify potential reactive metabolites leading to the irreversible (or quasi-irreversible) inhibition of CYPs.

The qualification of the structural alerts was conducted using the 3A4 TDI dataset and the statistical analysis (see Methods section, Structural alerts) led to the definition of a selection of alerts as being predictive. These alerts are listed in Table 4.

**TABLE 4:** MBI alerts qualified as statistically significant and predictive (based on the number of examples matching the alert).

Name		General representation of the structural alert	Description
Benzodioxoles (methylenedioxyphenyl compounds)		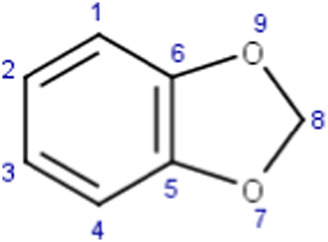 No substitution in C8	The benzodioxole moiety is included in the literature list of the main substructures causing MBI of cytochrome P450 (Correia, 2005; Fontana et al., 2005; Kalgutkar et al., 2007; Orr et al., 2012; Yu et al., 2015) Time-dependent inhibition of P450 by benzodioxole derivatives is proposed to involve P450 oxidation of the methylenedioxy ring carbon to a carbene intermediate (Kalgutkar and Soglia, 2005; Kalgutkar et al., 2012). This reactive metabolite may inhibit the enzyme by strongly coordinating to heme iron in the active site of P450 (Kalgutkar et al., 2005). An alternative pathway leading to MBI may also involve the formation of the o-quinone intermediates then binding to the P450 active site residues (Kalgutkar et al., 2012; Orr et al., 2012)
Furans		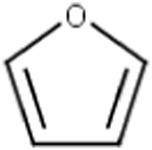	The furan moiety is included in the literature list of the main substructures causing MBI of cytochrome P450 (Fontana et al., 2005; Kalgutkar et al., 2007; Orr et al., 2012; Yu et al., 2015). MBI is associated with the formation of an epoxide intermediate that is susceptible to nucleophilic attack by nitrogen atoms of the CYP’s protein residues or of the CYP’s heme group. Formation of a covalent bond between the reactive metabolite and P450 results in inactivation of the enzyme (Fontana et al., 2005)
Terminal (omega and omega-1) alkynes		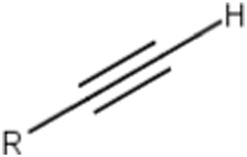 or 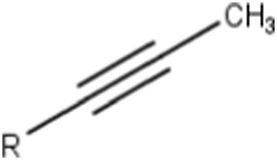 (R = carbon substitution)	Alkynes are in general included in the list of the main substructures causing MBI of cytochrome P450 (Correia, 2005; Fontana et al., 2005; Kalgutkar et al., 2007; Orr et al., 2012; Yu et al., 2015). The omega and omega-1 alkynes (terminal) are also specifically discussed as MBI structural alerts (36). The position of the triple bond seems to affect the bioactivation pathway and the type of reactive metabolites that may alkylate the heme prosthetic group or the apoprotein (Correia, 2005; Kalgutkar et al., 2005; Kalgutkar et al., 2007). It was noted that terminal acetylenes (RC≡H) form covalent adducts with the P450 heme group (Correia, 2005)
Primary amines (non-aromatic)		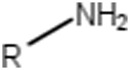 R = alkyl carbon (No additional substituents bound to N)	Primary amines are known to be quasi-irreversible CYP inactivators (Orr et al., 2012). In general, alkyl amines may cause MBI of cytochrome P450 and they are responsible for the majority of clinical drug-drug interactions (Yu et al., 2015). More specifically, amines can be oxidized and coordinate the heme iron inhibiting P450 (Correia, 2005; Kalgutkar et al., 2007). It is suggested that primary amines are first hydroxylated to hydroxylamines, and these can undergo further oxidation to a nitroso species, which can then coordinate the heme iron (Correia, 2005; Kalgutkar et al., 2007)
Secondary cyclic amines (non-aromatic)		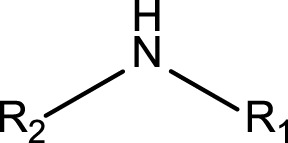 R_1_, R_2_ = alkyl carbon R_1_-N-R_2_ are part of a cycle	In general, alkyl amines may cause MBI of cytochrome P450 (Yu et al., 2015). More specifically, amines can form intermediates following oxidation and after this they can coordinate the heme iron and inhibit P450 (Correia, 2005; Kalgutkar et al., 2007). However, primary amines are required for the formation of the metal complex causing quasi-irreversible inhibition of P450 (Correia, 2005; Kalgutkar et al., 2007). Indeed, secondary cyclic amines are considered to alert for the formation of reactive metabolites via a nitroxide radical (Kalgutkar et al., 2005)
Cyclopropylamines		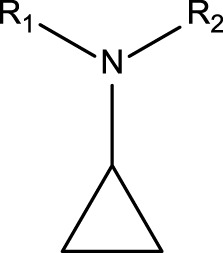 R_1_, R_2_ = H or carbon with no heteroatom attachement	Cyclopropylamines are well-known substructures causing MBI of cytochrome P450 (Kalgutkar et al., 2005; Yu et al., 2015). A bioactivation pathway of cyclopropylamines has not been fully elucidated. MBI is proposed to occur via covalent binding to the P450 active site or apoprotein (Kalgutkar et al., 2005; Orr et al., 2012). Cyclopropylamines with or without an abstractable alpha-carbon hydrogen can inactivate P450; cyclopropylanilines are reported not to inhibit P450 activity (Kalgutkar et al., 2005; Kalgutkar et al., 2007)
Hydroquinone (ortho and para) derivatives		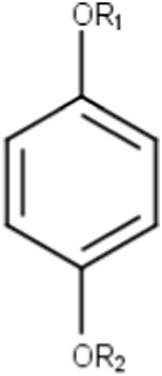 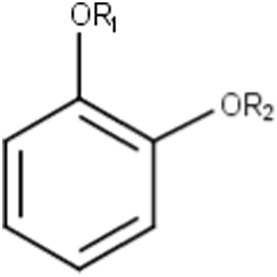 R_1_, R_2_ = H or saturated carbon 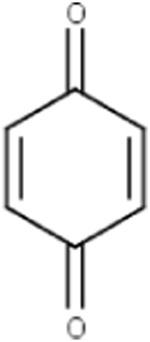 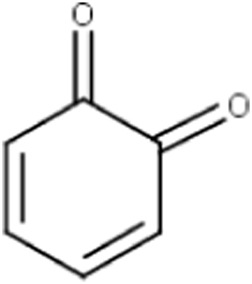	Phenols are generally included in the list of the main substructures causing MBI of cytochrome P450 (Orr et al., 2012; Yu et al., 2015). Following bioactivation of phenols, quinone intermediates and/or epoxides may be formed which are likely associated with MBI (Kalgutkar et al., 2007)
Epoxides		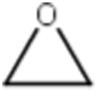	The epoxide moiety is included in the list of the main substructures causing MBI of cytochrome P450 (Fontana et al., 2005). They are electrophilic functional groups that can then directly react with nucleophiles contained in the biological macromolecules (Kalgutkar et al., 2012)
Piperazines (tertiary amines)		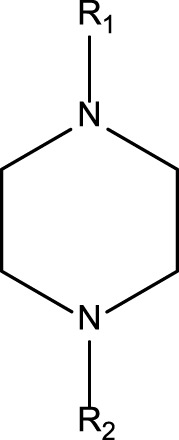	Piperazines are not explicitly mentioned in the literature as alerts for MBI of cytochrome P450. Bioactivation of the piperazine ring is mediated by P450 or monoamine oxidase, which catalyze reactions such as *N*-dealkylation, ring hydroxylation, *N*-oxygenation, and ring opening (Bolleddula et al., 2014). Bioactivation is proposed to involve hydroxylation at the alpha- or beta- carbon atom forming an unstable carbinolamine intermediate; This then leads to the formation of amino aldehyde and an iminium ion/imine that are reactive electrophile intermediates and that can covalently bind to nucleophiles contained in biological macromolecules (Bolleddula et al., 2014). For compounds containing the phenyl piperazine system, bioactivation may also occur via formation of a quinone-imine intermediate. This is formed following hydroxylation of the phenyl ring (Bolleddula et al., 2014)
Alkylphenols (ortho- and para), quinone-methide precursors		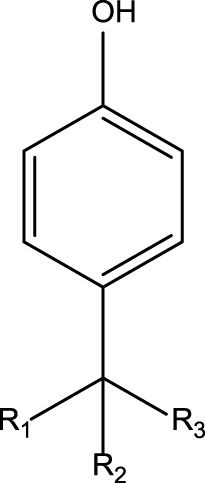 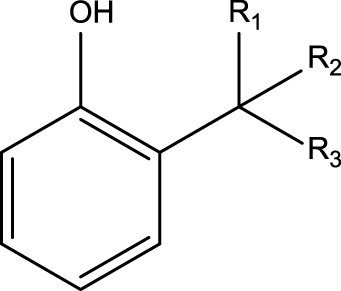 R_1_, R_2_, R_3_: H or C	In general, the phenol moiety is included in the list of the main substructures causing MBI of cytochrome P450 (Orr et al., 2012; Yu et al., 2015). Bioactivation of alkylphenols may lead to the formation of the reactive quinone-methide species (Kalgutkar and Soglia, 2005) causing apoprotein arylation in P450 (Orr et al., 2012)
Alkylaromatic ethers (ortho- and para), quinone-methide precursors		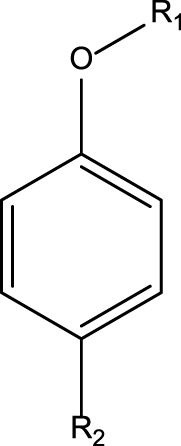 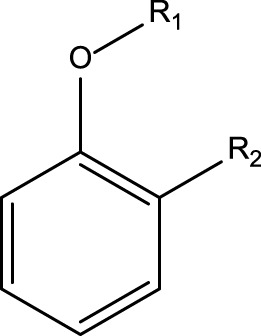 R_1_, R_2_: saturated carbon	Alkylaromatic ethers are not explicitly mentioned in the literature as alerts for MBI of cytochrome P450. Notably, the P450-mediated oxidative *O*-dealkylation reactions of *o*- or *p*-alkylaromatic ethers may form the corresponding phenols (Kalgutkar et al., 2005). Formation of reactive quinone-methide species can then occur (Kalgutkar and Soglia, 2005) causing apoprotein arylation in P450 (Orr et al., 2012)
Reactive arenes		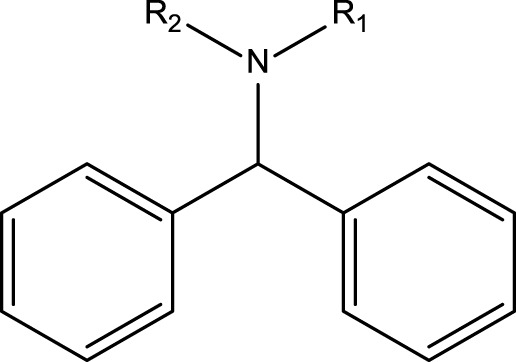 R_1_, R_2_ = H or C 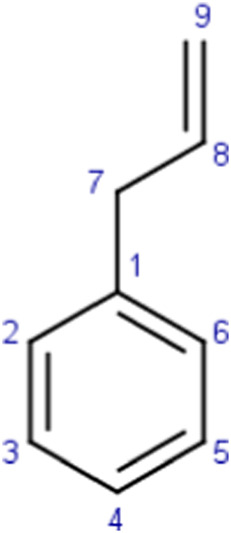 C9: no heteroatom attachment C7: saturated carbon	Arenes may potentially lead to reactive metabolites (i.e., arene oxides) upon epoxidation (Kalgutkar et al., 2005; Kalgutkar and Soglia, 2005). This specific alert identifies specific chemical environments containing the arene substructure and other moieties that are mentioned in the literature as being associated with MBI: alkyl amines (Correia, 2005; Kalgutkar et al., 2007; Orr et al., 2012; Yu et al., 2015) and alkenes (Correia, 2005; Orr et al., 2012; Yu et al., 2015)
Arenes (miscellaneous)		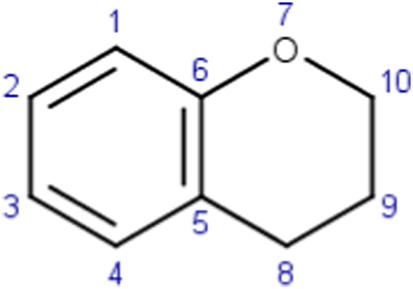 Bonds in the heteroaromatic rings can be of various types (e.g., single, double)	Arenes may potentially lead to reactive metabolites (i.e., arene oxides) upon epoxidation (Kalgutkar et al., 2005; Kalgutkar and Soglia, 2005). This specific alert identifies benzopyran analogues

### 3.5 Predictive performance of QSAR models and SAR profiler

The predictive performance statistics of CYP inhibition models using cross validation experiments and external validation experiments are presented in Table 5. Overall, the models offer 78%–84% sensitivity and 79%–84% negative predictivity in cross-validation. Concordance of the cross-validation and the external validation of the models range from 79% to 83% and from 66 to 86, respectively. Unlike training sets, the external validation sets are almost exclusively made of drugs. The difference in chemical space between the two databases (Figure 2 External validation database overview (A) Number of chemicals in each database. (B)) may explain relatively lower sensitivities for external validations. Additional validation studies were carried out to determine the optimal approach to combine CYP3A4 (MBI) predictions from SAR profiler and QSAR. Table 5 shows the performance statistics when a SAR profiler prediction is used to justify an overall positive or negative prediction only in cases where a QSAR prediction was equivocal.

**TABLE 5:** Statistical performance of CYP inhibition models.

Model	Cross-validation					External validation						
	3A4 QSAR	2C9 QSAR	2C19 QSAR	2D6 QSAR	3A4 (TDI) QSAR	3A4 QSAR	2C9 QSAR	2C19 QSAR	2D6 QSAR	3A4 (TDI) QSAR	3A4 SAR profiler	SAR profiler and 3A4 (TDI) QSAR
Sensitivity (%)	78	81	84	84	80	61	55	64	75	61	92	63
Specificity (%)	78	79	80	83	81	67	87	80	87	82	7	74
Pos Pred^a^ (%)	69 (79)	65 (78)	61 (80)	69 (82)	80	30 (65)	38 (80)	19 (72)	38 (83)	85	56	79
Neg Pred^a^(%)	86 (79)	89 (81)	93 (84)	92 (84)	81	88 (64)	93 (67)	97 (73)	97 (80)	56	40	56
Concordance (%)	79	80	81	83	81	66	83	79	86	69	55	67
Coverage (%)	82	85	86	86	89	74	71	70	77	68	61	72
Chi-squared	1760	1,123	833	1719	206	9	21	11	36	13	0.02	10

^a^ Numbers in parentheses show normalized predictivity.

## 4 Discussion

(Q)SAR models have been proven to be particularly useful for rapid screening of drug candidates during drug development. Additionally, they are used in a regulatory environment to provide rapid assessment of toxicological and pharmacological properties. Models that generate interpretable predictions are more desirable by regulators as supporting evidence on the relevance of a prediction can be more easily interrogated. Furthermore, application of complementary models has been proven to be successful in supporting the International Council on Harmonization (ICH) M7 guidance (Landry et al., 2019). In the present study, two modeling techniques have been utilized to construct CYP inhibition models. These models can facilitate identification of structural alerts and help determine whether a metabolite is to be investigated in reversible and time-dependent *in vitro* studies under the DDI guidance (Sudsakorn et al., 2020).

### 4.1 Interpretability of structural alerts

In contrast to “black box” models, the current work identifies molecular features responsible for CYP enzyme inhibition of drugs and other small molecules. As seen in Table 3, planar aromatic rings and secondary amines are among the top reversible inhibitory alerts for all CYPs. Earlier studies found that strong binding properties of amines and nitrogen-containing, heterocyclic derivatives intensify their inhibitory activities (Testa and Jenner, 1981). In addition, scaffolds containing oxygen or sulfur atoms attract iron cation of cytochrome P-450 through electronic interactions, resulting in 6-coordinated complexes (Testa and Jenner, 1981).

The presence of aromatic rings among top structural alerts for CYP3A4 inhibition may be due to pi–pi stacking interactions between those rings and phenylalanine residues uniquely present in the CYP3A4 (Beck et al., 2021). Also, the presence of flexible backbones allows a ligand to better fit into the active site of CYP3A4 (Kaur et al., 2016). That may explain the top linear structural alerts for CYP3A4 inhibition.

All top features for CYP2D6 have amine groups, in agreement with previous findings (Sridhar et al., 2012). The active site of CYP2D6 contains two carboxylic acids from Q216 and D301 that are involved in substrate recognition and binding (de Groot et al., 2009; Wang et al., 2009). These acidic residues interact with primary and secondary amines present in the inhibitors (Beck et al., 2021). The presence of a basic nitrogen atom and a planar aromatic ring in many of CYP2D6 substrates has been reported before (Yu et al., 2004; Gay et al., 2010). Furthermore, aromatic rings in inhibitors may interact with F120 and F483 through pi–pi stacking (Beck et al., 2021).

The presence of an amine residue has been proposed to favor substrate binding to CYP2C9 through electrostatic interactions (Jones et al., 1996). Heterocycles and heterocyclic nitrogen atoms that are frequently seen in CYP2C19 inhibitors coordinate with Heme-501 (Beck et al., 2021). Also, numerous pi–pi stacking interactions occur between small-molecule arylene groups and F114 and F476 in CYP2C19 (Beck et al., 2021). Similar pi–pi stacking interactions are observed in CYP2C9.

On the other hand, tertiary aromatic amines such as pyridine are among features with lowest Z-scores for all models. Carboxylic acid is in the bottom of the list for CYP3A4 (both RI and TDI) and CYP2D6.

Among structural features for CYP3A4 TDI, cyclopropylamine is reported to cause mechanism-based inhibition of CYP enzymes through heme alkylation (Orr et al., 2012; Hoemann et al., 2016; Xu et al., 2022). Experiments show that pyridine is a weak inactivator of CYP3A4 due to the weak ligation of its nitrogen to the heme iron (Kaur et al., 2016).

Among 58 MBI alerts obtained from literature, 24 alerts are similar to, or exact matches of the alerts identified by the QSAR model. Cyclopropyl amines, hydroquinone derivatives, alkoxy benzenes, and heteroaromatic rings are the overlapping alerts that cover the largest subset of compounds in the TDI training set with up to 253 common compounds in the training set.

These results suggest that the two modeling techniques can be used in combination to obtain additional weight of evidence for the MBI predictions.

### 4.2 Practical application

An external validation set was used to evaluate the predictive performance of the RI QSAR models and to determine the optimal approach for combining predictions from the current SAR profiler for MBI and QSAR model for CYP3A4 TDI. Previous studies showed that using multiple (Q)SAR models in combination can increase the sensitivity when a positive prediction from any one system is used to justify an overall positive prediction (Hillebrecht et al., 2011; Contrera, 2013; Sutter et al., 2013; Faramarzi et al., 2022). However, it should be noted that the SAR profiler has been constructed to provide information on potential mechanisms of inhibition for all CYP enzymes while the QSAR model and the external validation set are both based only on the CYP3A4 TDI data. As such, the rules for using two methodologies in combination have been adjusted. Overall, it was found that the SAR profiler identified additional positive signals related to other CYP enzymes and therefore showed high sensitivity and low specificity in the external validation. However, in the cases where an equivocal prediction was generated by the CYP3A4 TDI QSAR model, a positive prediction from the SAR Profiler provides additional mechanistic information to determine the relevance of the positive and negative features obtained from the QSAR model prediction. Moreover, it was determined that sensitivity can be increased to 69% (Table 5) without drastically decreasing specificity when a positive prediction from the SAR profiler is used to justify an overall positive prediction when the QSAR prediction was equivocal.

## 5 Conclusion

In the present study, an extensive literature search was performed and alerts for mechanism-based inhibition of CYPs were collected and used to develop a SAR profiler. Furthermore, five quantitative structure-activity relationship (QSAR) models were constructed to predict not only time-dependent inhibition of the major drug-metabolizing enzyme, CYP3A4, but also reversible inhibition of CYPs 3A4, 2C9, 2C19 and 2D6. Structural alerts identified by these QSAR models and the SAR profiler were compared and although some overlap was identified, each model was found to contain additional information. Lastly, an optimal method for combining predictions from the different methodologies was determined. These new models provide a faster and more effective evaluation of CYP inhibition potential and may be used to support of DDI structural alert identification.

## Data Availability

The datasets presented in this study can be found in online repositories. The names of the repository/repositories and accession number(s) can be found in the article/Supplementary Material.
