# Supplementary material for: Novel (Q)SAR models for prediction of reversible and time-dependent inhibition of cytochrome P450 enzymes
Source: Front Pharmacol. 2025 Feb 12;15:1451164. doi: 10.3389/fphar.2024.1451164 (PMC11860084; doi:10.3389/fphar.2024.1451164)
Supplement: Supplementary file 1 [file DataSheet1.zip › Supplementary Material/Supplementary_TableS2.DOCX]

# Supplementary Table S2:

The 58 MBI alerts as collated from the literature review. Representative structures of the alert chemical space are shown.

| **Alert Name** | **Representative structures** |
| --- | --- |
| Benzodioxoles (methylenedioxyphenyl compounds) | 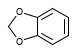 |
| Furans | 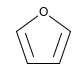 |
| Thiophenes | 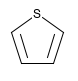 |
| Alkenes | 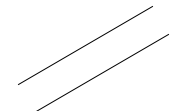 |
| Terminal alkenes | 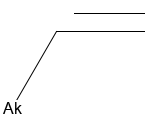 |
| Alkenes, conjugated derivatives | 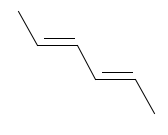 |
| Alkynes | 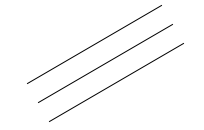 |
| Terminal (omega and omega-1) alkynes | 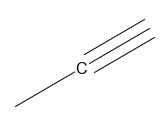 |
| 2-Alkylimidazole derivatives | 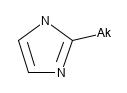 |
| Amines (non-aromatic) | 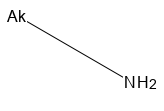 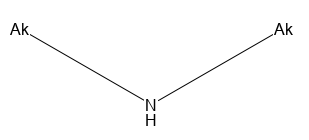 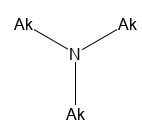 |
| Primary amines (non-aromatic) | 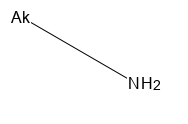 |
| Secondary amines (non-aromatic) | 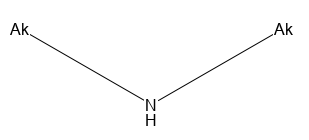 |
| Secondary cyclic amines (non-aromatic) | 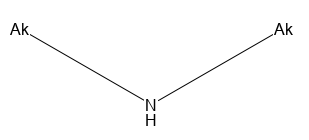 (cyclic) |
| Tertiary amines (non-aromatic) | 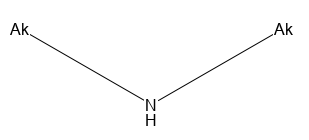 |
| Tertiary cyclic amines (non-aromatic) | 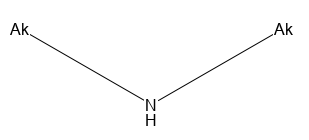 (cyclic) |
| Cyclopropylamines | 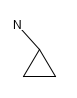 |
| Thiazolidinedione derivatives | 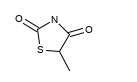 |
| 4-Alkyl-1,4-dihydropyridines | 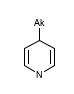 |
| Dithiocarbamates | 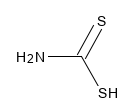 |
| Alkylpyrrole and alkylindole derivatives | 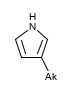 |
| Aniline derivatives | 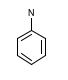 |
| Aminophenols derivatives (ortho- and para-) | 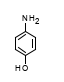 |
| Alkyl aniline derivatives (ortho- and para-) | 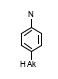 |
| Dianiline derivatives (ortho- and para-) | 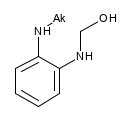 |
| Thiazoles and 2-amino derivatives | 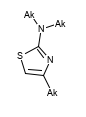 |
| 5-Aminooxindole derivatives | 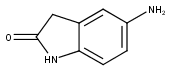 |
| Dihaloalkanes | 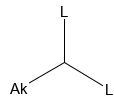 L = halogen |
| Phenols | 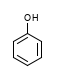 |
| Alkylphenols (ortho- and para), quinone-methide precursors | 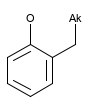 |
| Hydroquinone (ortho and para-) derivatives | 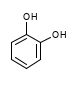 |
| Hydrazines/hydrazides (mono-, di-substituted) | 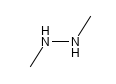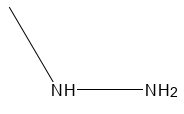 |
| Hydrazine analogs | 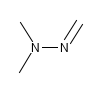 |
| Isothiocyanates | 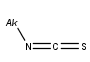 |
| Thiols | 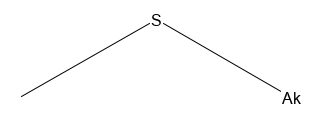 |
| Allylsulfides and allylsulfones | 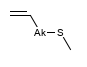 |
| Dichloro- and Trichloro-ethylenes | 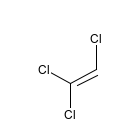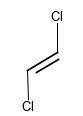 |
| Epoxides | 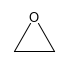 |
| Alkenes, alpha,beta-unsaturated carbonyl compounds | 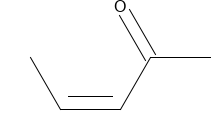 |
| Alkenes, alpha,beta-unsaturated carboxylate compounds | 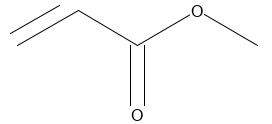 |
| Alkynes (functional groups) | 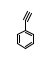 |
| Piperazines (tertiary) |  |
| Thiazoles |  |
| Alkyl aniline derivatives (ortho- and para-) (secondary) | 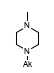 |
| Alkylaromatic ethers (ortho- and para), quinone-methide precursors | 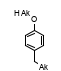 |
| Carboxylic acids (alkyl) | 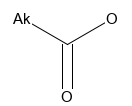 |
| Carboxylates (alkyl esters) | 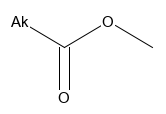 |
| Disulfides | 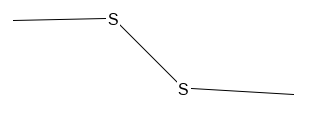 |
| Triazoles | 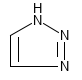 |
| Heteroaromatic amines (primary) | 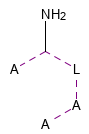A= any atom but H; L = N,O, S; Dashed bonds: aromatic bonds |
| Heteroaromatic amines (secondary) | 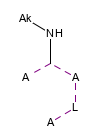A= any atom but H; L = N,O, S; Dashed bonds: aromatic bonds |
| Heteroaromatic amines (tertiary) | 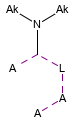A= any atom but H; L = N,O, S; Dashed bonds: aromatic bonds |
| Arenes (reactive features - MBI) | 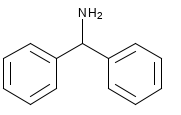 |
| Arenes (miscellaneous) | 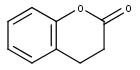 |
| Aniline derivatives (secondary) | 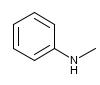 |
| Aniline derivatives (sulfonyl) | 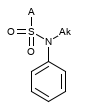A = any atom but H |
| Heteroaromatic rings | 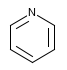 |
| Styrene derivatives | 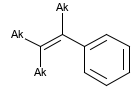 |
| Pyrrolidines (tertiary) | 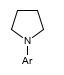 |
